# Emergency Care Sensitive Conditions in Brazil: A Geographic Information System Approach to Timely Hospital Access

**DOI:** 10.1016/j.lana.2021.100063

**Published:** 2021-09-10

**Authors:** Julia Elizabeth Isaacson, Anjni Patel Joiner, Arthi Shankar Kozhumam, Nayara Malheiros Caruzzo, Luciano de Andrade, Pedro Henrique Iora, Dalton Breno Costa, Bianca Maria Vissoci, Marcos Luiggi Lemos Sartori, Thiago Augusto Hernandes Rocha, Joao Ricardo Nickenig Vissoci

**Affiliations:** 1 Duke University School of Medicine, DUMC 3170, Durham, North Carolina, 27710, United States of America; 2 Duke Global Health Institute, 310 Trent Drive, Durham, North Carolina, 27710, United States of America; 3 Division of Emergency Medicine, Department of Surgery, Duke University Medical Center, 2301 Erwin Road, Durham, North Carolina, 27710, United States of America; 4 Department of Physical Education, State University of Maringá, Av. Colombo, 5790 - Zona 7, Maringá - Paraná, 87020-900, Brazil; 5 Department of Medicine, State University of Maringá, Av. Colombo, 5790 - Zona 7, Maringá - Paraná, 87020-900, Brazil; 6 Department of Psychology, Federal University of Health Sciences of Porto Alegre, R. Sarmento Leite, 245 - Centro Histórico, Porto Alegre - Rio Grande do Sul, 90050-170, Brazil; 7 Program for Health Sciences, State University of Maringá, Av. Colombo, 5790 - Zona 7, Maringá - Paraná, 87020-900, Brazil; 8 Department of Computer Science, Pontifical Catholic University of Rio Grande do Sul, Av. Ipiranga, 6681 - Partenon, Porto Alegre - Rio Grande do Sul, 90619-900, Brazil

**Keywords:** Emergency Care Sensitive Conditions, Access to care, Geographic Information System, Brazil, Emergency Medicine, Emergency Care, Trauma, Stroke, Heart Attack, STEMI

## Abstract

**Background:**

The benefits of treatment for many conditions are time dependent. The burden of these emergency care sensitive conditions (ECSCs) is especially high in low- and middle-income countries. Our objective was to analyze geospatial trends in ECSCs and characterize regional disparities in access to emergency care in Brazil.

**Methods:**

From publicly available datasets, we extracted data on patients assigned an ECSC-related ICD-10 code and on the country’s emergency facilities from 2015-2019. Using ArcGIS, OpenStreetMap, and WorldPop, we created catchment areas corresponding to 180 minutes of driving distance from each hospital. We then used ArcGIS to characterize space-time trends in ECSC admissions and to complete an Origin-Destination analysis to determine the path from household to closest hospital.

**Findings:**

There were 1362 municipalities flagged as “hot spots,” areas with a high volume of ECSCs. Of those, 69.7% were more than 180 minutes (171 km) from the closest emergency facility. These municipalities were primarily located in the states of Minas Gerais, Bahia, Espiríto Santo, Tocantins, and Amapá. In the North region, only 69.1% of the population resided within 180 minutes of an emergency hospital.

**Interpretations:**

Significant geographical barriers to accessing emergency care exist in certain areas of Brazil, especially in peri-urban areas and the North region. One limitation of this approach is that geolocation was not possible in some areas and thus we are likely underestimating the burden of inadequate access. Subsequent work should evaluate ECSC mortality data.

**Funding:**

This study was funded by the Duke Global Health Institute Artificial Intelligence Pilot Project.

**Research in Context**

**Evidence before this study**

Emergency care sensitive conditions (ECSCs) are conditions for which rapid symptom identification and timely emergency care can have a mortality and/or morbidity benefit. Adequate, appropriate access to emergency care services is essential in avoiding preventable mortality due to ECSCs. The burden of emergency disease is particularly high in low- and middle-income countries. About half of the municipalities in Brazil cannot easily access a network providing emergency care and several municipalities exist in which inhabitants reside more than 60 kilometers from emergency care.

**Added value of this study**

Geographic information system (GIS) approaches are often used for conducting spatial analyses of complex health disparities. Existing literature applying GIS to emergency care in Brazil focuses on locations of health facilities but not on relevant street networks. This study uniquely applies GIS to characterize regional disparities in access to emergency care in Brazil by analyzing the geospatial distribution of ECSCs in relation to health facilities via existing roads. Our findings demonstrate significant geographical barriers to accessing emergency care in Brazil. The literature well describes the presence of access to care barriers in the North, but our results reveal that other parts of the country, particularly peri-urban areas, also experience geographical challenges in accessing emergency care.

**Implications of all the available evidence**

We believe our work will be helpful to policymakers and health authorities by informing discussions on addressing region-specific geographical access to care barriers. Resultantly, we hope our findings will support public health efforts aimed at improving the ability of Brazil's emergency care network to provide prompt emergency treatment to patients whose conditions most depend on it. Our approach can also be used in other countries and applied in various contexts to assist in making evidence-based policy decisions. Future work should evaluate ECSC mortality and morbidity data to gain further insight into the effects of the inaccessibility of emergency care in much of Brazil.

Alt-text: Unlabelled box

## 1 Introduction

Many medical conditions exist for which immediate treatment is essential in achieving favorable health outcomes. Worldwide, in 2015, emergency conditions resulted in 28•3 million deaths, accounting for 50•7% of total deaths [1]. Disease states for which rapid symptom identification and expeditious emergency care improve morbidity and/or mortality are deemed emergency care sensitive conditions (ECSCs) [2].

Adequate access to emergency care is essential in avoiding preventable deaths and losses [3]. This is particularly true in the treatment of ECSCs in low and middle-income countries (LMICs) where barriers to accessing the healthcare system are often the greatest [3,4]. Of the world's health-related emergencies, 90% occur in LMICs [5].

In Brazil, at both the municipality and the regional levels, striking disparities exist in the availability of healthcare, including emergency care [3,^6^,7]. Previous work has demonstrated that approximately half of the country's municipalities cannot easily access an emergency care network and there are several municipalities in which inhabitants live more than 60 kilometers away from emergency care [3,7]. Those living in rural communities and the Amazon region are disproportionately affected [3]. Brazil's Mobile Emergency Medical Service, SAMU, with its focus on the decentralization of ambulance bases, is one approach being used to increase access to emergency care for inhabitants in remote areas. However, geographical barriers to accessing emergency services exist not only in rural portions of the country, but also in regions close to urban areas [3,^7^,8].

There are many markers of access to care, with geography being particularly important [9]. The existing literature on geographical barriers to emergency care in Brazil focuses mainly on emergency care distribution as it relates to morbidity and mortality, utilizing distributions of patient income and education rather than relevant transportation networks [3]. The aim of the present work is to use a GIS approach to characterize regional disparities in access to emergency care in Brazil by analyzing (1) the trends in the geospatial distribution of ECSCs and (2) the distance to the closest emergency hospital. We hope the results of this study will support discussions on designing health policies to ensure better availability of emergency services in Brazil, improving the ability of Brazil's emergency care network to provide prompt treatment to patients whose conditions most depend on it.

## 2 Methods

### 2.1 Study design

We conducted a longitudinal, ecological study to analyze the geospatial distribution of ECSCs and access to emergency care in Brazil from 2015 to 2019. We collected publicly available data on the 10,279,441 patients assigned an ICD-10 code associated with an ECSC as well as on the country's approximately 2,900 emergency medicine (EM) facilities [10]. To generate evidence capable of supporting health policy discussions addressing a lack of access to emergency care, we performed a sequence of steps to represent the current situation in Brazil. First, we assessed the distribution of health facilities capable of providing emergency care and the population with access to these health services. In the second step, we performed geolocation of the ECSC hospital admissions and determined time-space clustering patterns. In the final step, we built origin-destination matrices to find the path from ECSC to the closest emergency facility via existing street networks. The results from the final step were combined with data gathered in the previous two steps to determine the location of areas with both a high number of ECSCs and geographical barriers to access.

### 2.2 Study setting and population

#### 2.2.1 Study setting

Brazil is the largest country in South America in both size and population. The country spans over 8•4 million square kilometers and is home to more than 211 million inhabitants [11,12]. Brazil is made up of five regions: North, Northeast, Midwest, South, and Southeast. The country has 26 states and one federal district which are subdivided into 5,565 municipalities. The municipalities are federal entities with autonomous governments and are not merely subdivisions of the state [13]. The population of the municipalities are wide-ranging. As of 2015, 17 municipalities were home to more than one million people (22% of the population) and 44% of the municipalities were home to fewer than 10,000 people (6•3% of the population)[13]

Brazil is an upper middle-income country; however, the wealth is not distributed equally. Rather, Brazil is amongst the most unequal countries in the world [14]. The population is covered by The Unified Health System (Sistema Único de Saúde, SUS), the country's publicly funded, decentralized, and universal health care system with an emphasis on primary care as the entry point into the system. Foundational to the institution of the SUS was the constitution's emphasis that health is a right of citizens and a duty of the state to provide [15]. The SUS, enacted in 2003, considerably increased access to health care, predominantly due to the larger healthcare workforce and increased primary care services. In 1981, 8% of Brazilians had received health services in the past 30 days. In 2008, this number had increased to 14•2%. Currently, the SUS provides health services in all states and municipalities although problems persist with the equitability of access [15].

#### 2.2.2 Study population

Characterization of Brazilian emergency care services was accomplished using data from the National Registry of Health Facilities (CNES), a registry of all Brazilian facilities linked to the Unified Health System [7,16]. From the initial list of 350,000 facilities, hospitals with emergency capabilities were selected. A hospital was considered as having emergency capabilities if it was categorized as either a general hospital, specialized hospital, general emergency department, mixed unit, or emergency unit and had more than 50 inpatient (including ICU) beds. In Brazil, hospitals with less than 50 beds are considered small; subsequently, the limited funding they receive makes provision of emergency care difficult [3,7]. These five categories of healthcare facilities, while heterogenous in their abilities, were selected based on the Ministry of Health's categorization scheme, deeming them capable of performing emergency care services. Similar numbers of EM hospitals were identified each year for further analysis (Table 1). The selected hospitals were then geolocated.

**Table 1:** Number of Brazilian Emergency Hospitals by Year, 2015-2019.

**Year**	**Hospitals (n)**
2015	2,935
2016	2,906
2017	2,930
2018	2,941
2019	2,926

To characterize the ECSC admissions, we extracted data from the Hospital Information System of the Brazilian Unified National Health System (SIH-SUS). The admissions selected for analysis were associated with at least one of the ICD-10 codes specified in Vashi et al.’s comprehensive list of 4,054 ECSC-related codes [17]. In total, 10,279,441 admissions were identified from 2015 to 2019 (Table 2). Of these admissions, 9,636,158 (94%) were geolocated following the methodology designed by Rocha et al (Supplementary Materials, Tables 1,2,&3) [10]. The address geolocated for each admission was the home address reported by the patient. The 643,283 admissions not accounted for were located mostly in urban areas (603,868) where the zip code could not be precisely geocoded (Supplementary Materials, Tables 1&2). The region with the largest number of admissions not geolocated was the Southeast region where the location of 446,093 (11%) ECSCs could not be determined (Supplementary Materials, Table 3). The Brazilian Institute of Statistics and Geography (IBGE) was used to obtain estimates of Brazil's total population as well as population by region and municipality so that adjusted rates weighted by population could be calculated [18].

**Table 2:** Number of Brazilian ECSCs by Year, 2015-2019.

**Year**	**ECSCs (n)**	**ECSCs Geocoded (n)**
2015	2,065,781	1,847,930
2016	2,013,545	1,790,327
2017	2,031,449	1,979,511
2018	2,069,733	2,004,113
2019	2,098,933	2,014,277
Total	10,279,441	9,636,158

**Table 3:** Data Sources Used and Composite Variables Extracted.

**Data Source**	**Variable(s) Extracted**
**Hospital Information System** (SIH-SUS)	Number of admissions Costs associated with each admission Latitude & longitude of patient households Length of hospital stay Procedures performed during hospitalization Type of bed associated with admission ICD-10 code Gender Age Municipality of residence
**Brazilian Institute of Statistics and Geography** (IBGE)	Country and city population estimates
**National Registry of Health Facilities** (CNES)	Number of EM facilities by year Latitude and longitude of EM facilities Type of facility Number of beds available by type Municipality in which hospital is located
**OpenStreetMap**	Transportation network (roads, railroads, ferries)
**WorldPop**	Dasymetric population, 2020

### 2.3 Data sources and variables

Data for this manuscript were collected from publicly available databases. National data on patient ECSC, gender, socioeconomic status, education level, race, marital status, and age were collected and aggregated at the municipality level. From the CNES, we extracted data on the characteristics of the facilities with emergency capabilities and their locations. Data to describe the population and determine the rate of events by year was obtained from the Brazilian Institute of Statistics and Geography. The WorldPop dataset comprises the population estimate from satellite imagery [19].

### 2.4 Data analysis

To further characterize the Brazilian emergency care network and its association with ECSC hospitalizations, three analytical steps were performed. First, we assessed the emergency care coverage weighted by population within a driving distance time threshold of 180 minutes. The purpose of this step was to identify regions where there is a lack of access to emergency care in Brazil. Depending on the population distribution and overlap with ECSC admissions, these regions may or may not be priorities to policy makers aiming to improve emergency care access. Secondly, we used ArcGIS to analyze the distribution of ECSCs from a time and space perspective. The clustering patterns obtained highlight regions historically presenting with high levels of ECSCs. Areas where such clusters also experience a lack of care can be said to be facing disproportional geographical access barriers. Lastly, we created an Origin-Destination Matrix using known street networks to determine the distance between the 9•6 million geolocated ECSC and the closest EM hospital with emergency capabilities. The distance measurement helps by attributing a magnitude to the geographical barrier. The creation of a metric to categorize the different levels of geographic barriers is a relevant proxy in the determination of priorities by policy makers and can help in discussions regarding the location-allocation of health resources.

### 2.5 First analytical step: assessment of regions facing a lack of emergency care coverage

To determine the size of the population within 180 minutes driving distance of the selected emergency centers, we created customized catchment areas. For each EM hospital, we built a service area based on the driving time to reach the facility. The road networks were obtained from the OpenStreetMap initiative [20]. Using the Network Analyst Extension of ArcGIS Pro 2•5, all possible pathways to the facility up to the limit of 180 minutes driving time were considered [21]. One hundred and eighty minutes was used as a threshold in accordance with the Lancet Commission on Global Surgery's indicators for surgical system strength [22]. This amount of driving time is approximately equal to 171km.

The result of this analytical step was non-overlapping polygons representing the catchment area of each facility offering emergency services in Brazil. To estimate the number of inhabitants within each polygonal catchment area, we overlapped the dasymetric population obtained from WorldPop [19]. Dasymetric population estimates are determined by analyzing data derived from satellite imagery, such as the presence of lights at night and the refraction of sunlight by artificial constructions, and spatial covariates using artificial intelligence algorithms [23]. The WorldPop file containing the distribution of the dasymetric population was analyzed using ArcGIS Pro's Zonal Statistics Tool, which calculates the sum of the pixel values corresponding to the population per square kilometer [24]. We chose to use the dasymetric population estimate, last updated in 2020, for the coverage analysis because the 2010 census is outdated and the 2020 census was postponed due to the COVID-19 pandemic. Further, the IBGE population estimate does not offer the geospatial granularity that our approach requires. At the end of this process, it was possible to identify areas not covered by emergency services, considering a 180-minute displacement threshold. Citizens in the regions outside the catchment areas face geographical challenges in reaching health facilities with emergency capabilities.

### 2.6 Second analytical step: time-space clustering analysis of ECSC admissions

We then further analyzed the time-space distribution of ECSC admissions using ArcGIS's Emerging Hot Spot Analysis [25]. The Emerging Hot Spot Analysis creates a 3-D representation of the ECSC rate by municipality with each layer representing one point in time [8]. Next, the tool calculates the Getis-Ord Gi* statistic for each layer using the provided Conceptualization of Spatial Relationship values [26]. A z-score, p-value, and hotspot bin classification is assigned to each layer [27]. Next, rank correlation analysis is done to determine the Mann-Kendall statistic [25]. Finally, the tool assigns each outcome to one of 16 designations [25]. Within these 16 possibilities, eight represent hot spot trends and eight represent cold spot trends. Hot spots are regions in which the ECSC rate by municipality is increasing. Cold spots represent the opposite trend. The result of this step highlighted the areas in the country historically facing a high volume of ECSCs. By overlapping these areas with those facing lack of access, as determined in the first step, portions of the country that should be prioritized when designing interventions to improve access to emergency care can be identified.

### 2.7 Third analytical step: origin-destination matrix

To better characterize any geographical barriers to accessing emergency care, we then used ArcGIS to perform an Origin-Destination (OD) Cost Matrix Analysis. This tool determines the shortest distance via street network between multiple origins and destinations [28]. Using this approach, we determined the distance in time and kilometers from the place of residence of the patient associated with each ECSC and the closest EM facility. If the travel time was greater than three hours, which is approximately equal to 171km, the admission was deemed as having no timely access. This analytical step was done to attribute a magnitude to the lack of access problem. Being aware of priority regions and having a metric capable of characterizing the size of the challenge creates optimal conditions for supporting health policy discussions regarding overcoming barriers to accessing emergency services.

### 2.8 Role of the Funding Source

This study was funded by the Duke Global Health Institute Artificial Intelligence Pilot Project. This funding source had no role in study design, in the collection, analysis, or interpretation of data, in the writing of the report, or in the decision to submit the paper for publication.

## 3 Results

The catchment area analysis, our first analytical step, demonstrated that a high percentage of the Brazilian population (95•3%) resides within 180 minutes of a health facility with emergency capabilities. Despite this general trend, the regional analysis revealed a range in percentage of the population covered within the time threshold. In the North, only 69•1% of the population resides within 180 minutes of an EM hospital. Approximately 93% of the population is covered in the Midwest and Northeast. In the South and the Southeast, over 99% of the population resides within the recommended time threshold.

**Figure 1:**
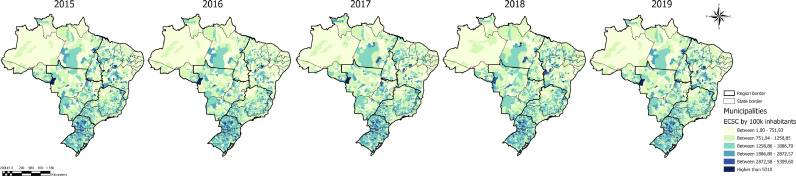
Emergency Care Sensitive Conditions (ECSCs) Rates by Population by Brazilian Municipality, 2015-2019.

The distribution of ECSCs among the Brazilian regions is heterogenous (Figure 1). The North and Northeast regions had fewer municipalities with high rates of ECSCs. The South was the region with the highest proportion of municipalities presenting with high rates of ECSCs. There were no obvious trends in ECSCs across the Brazilian regions over time from 2015 to 2019.

**Figure 2:**
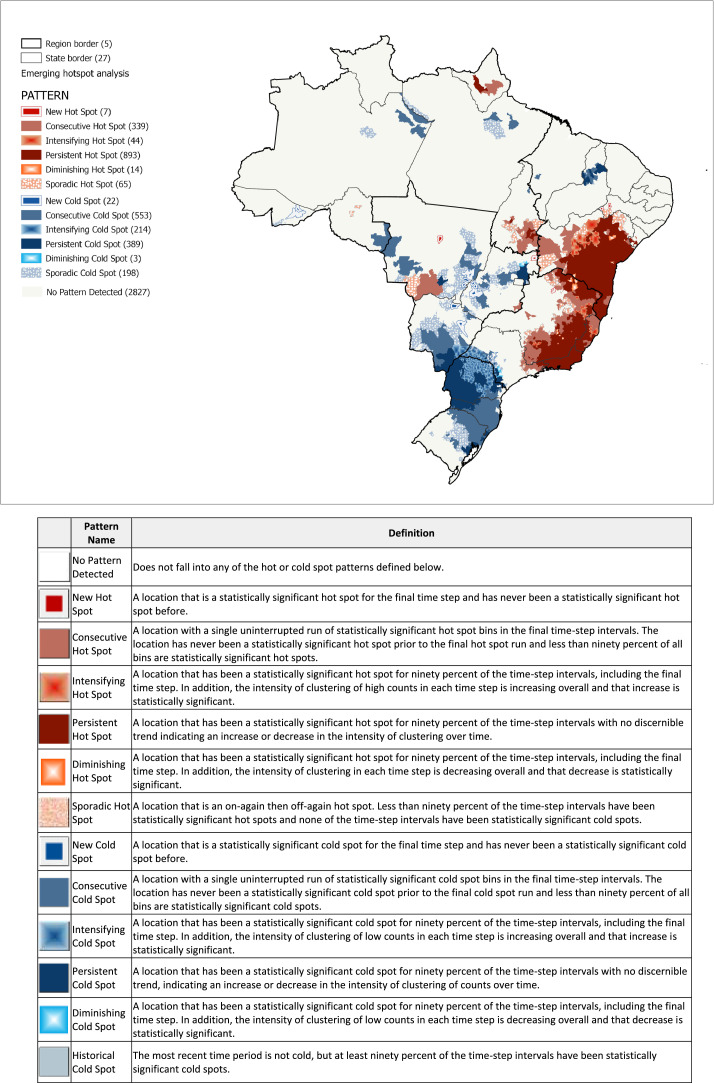
ECSC Emerging Hot Spot Analysis.

The Emerging Hot Spot Analysis of the ECSC rates revealed large clusters covering the South, Southeast, Northeast, and Midwest regions of the country (Figure 2). The blue colors seen largely in the South and portions of the Midwest represent municipalities with a decreasing trend in ECSCs from 2015 to 2019. The red colors seen in the Northeast and Southeast represent areas with an increasing trend in ECSCs. The general results of the space time trend analysis call attention to an uptrend of ECSC admissions in the most populated areas of the country as well as a heterogenous decreasing case trend in the South region.

**Figure 3:**
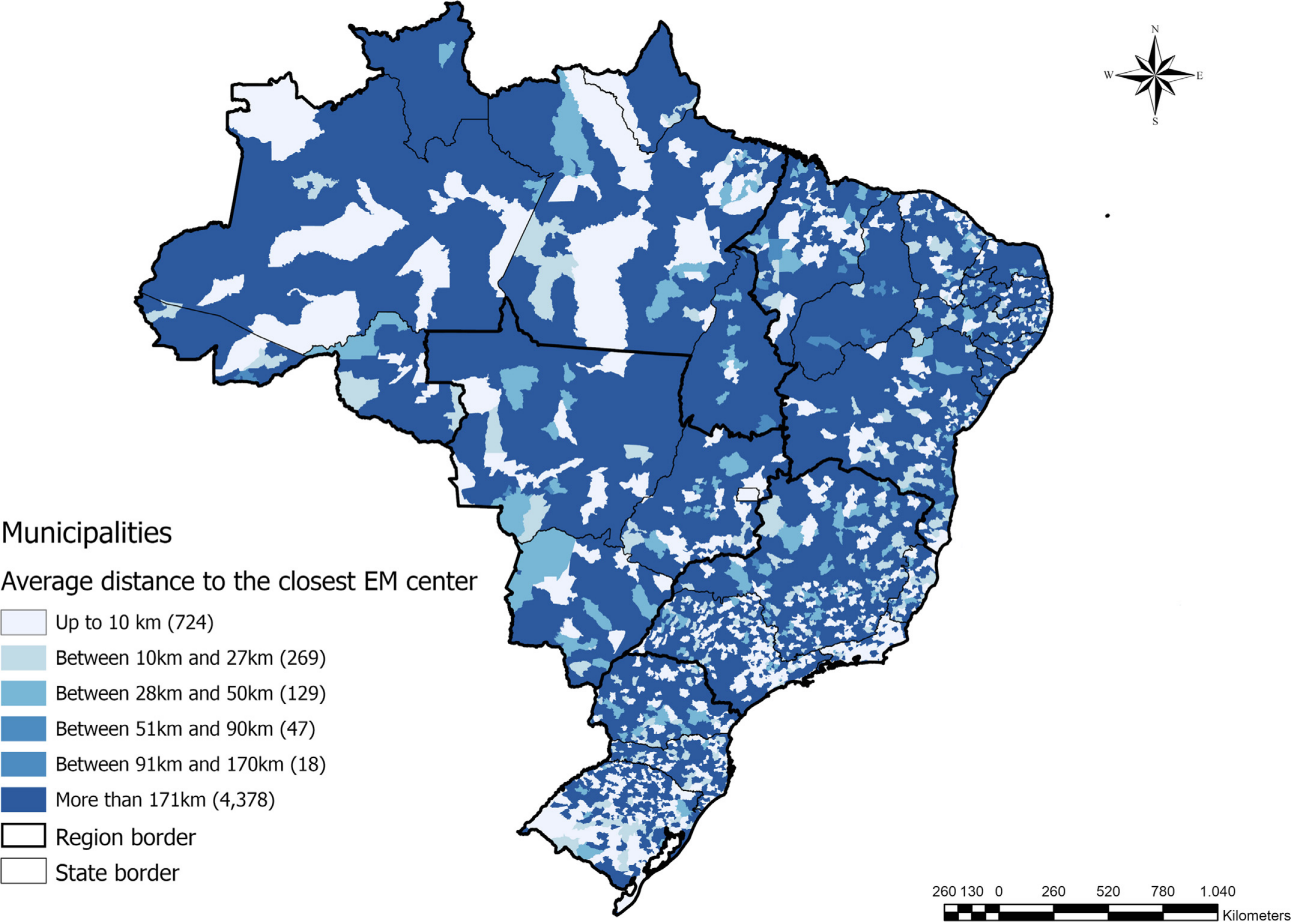
Average Distance Between Household and Closest Emergency Medicine (EM) Center.

Access to emergency care services using existing transportation networks in Brazil is heterogenous (Figure 3). Of the 5,565 Brazilian municipalities, 4,378 (78•7%) are more than 171 kilometers or 180 minutes away, on average, from a facility capable of performing satisfactory emergency care. Most of these municipalities are within rural areas in the North and Midwest regions of the country. In contrast, in the South and Southeast, most municipalities are within 170km of emergency care services, with many municipalities being within 10km.

**Figure 4:**
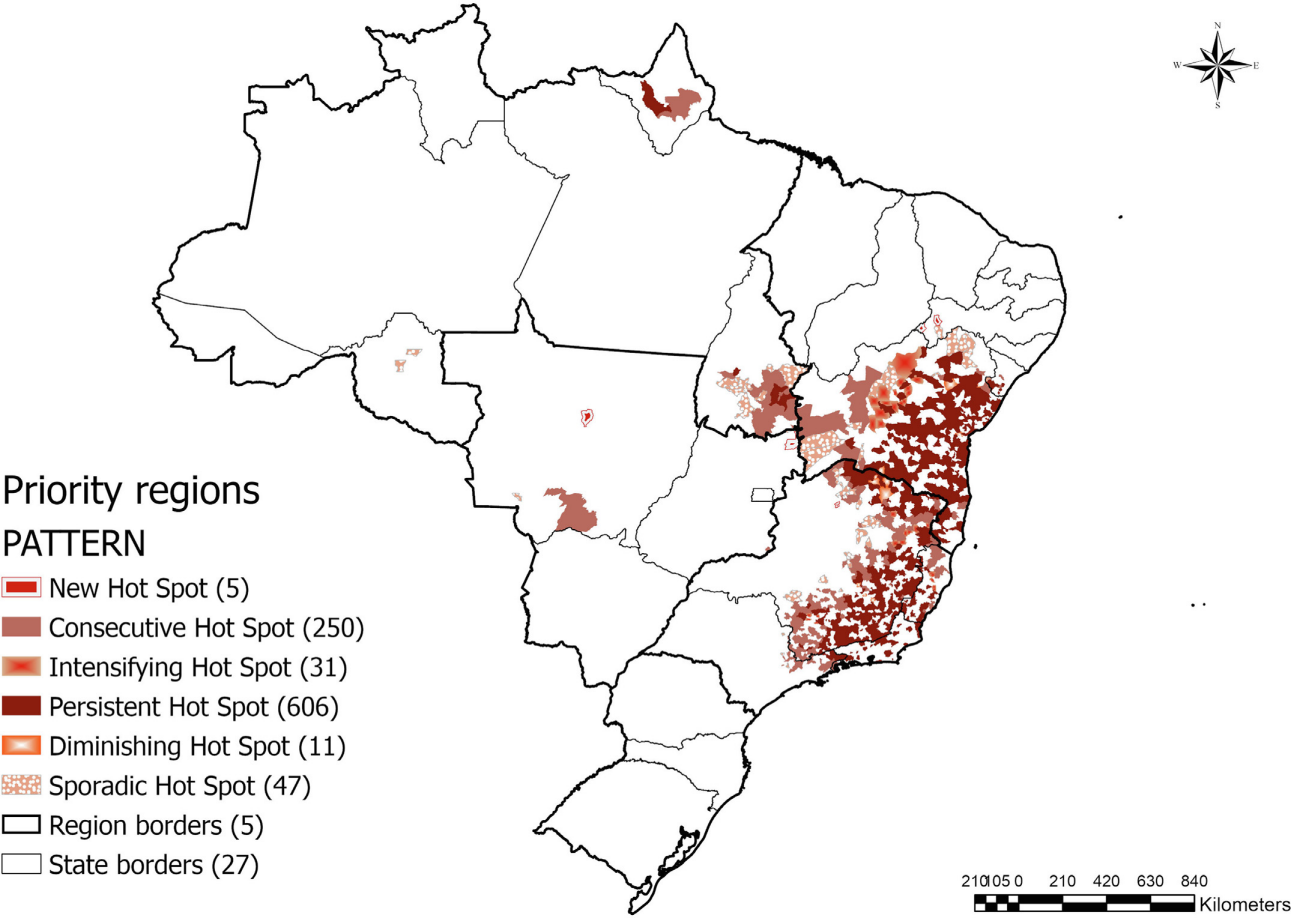
Areas with High Volume of ECSC Admissions Facing Geographical Access Barriers.

Figure 4 depicts areas that are experiencing both geographical access barriers to emergency care and an increasing burden of ECSC admissions. Of 1362 municipalities flagged as hotspots, 950 (69•7%) are more than 180 minutes of driving distance from the closest emergency facility. Large portions of the states of Minas Gerais, Bahia, Espiríto Santo, Tocantins, and Amapá face this challenge.

## 4 Discussion

The existing literature on access to emergency care in Brazil is limited. To our knowledge, this is the first study in the country describing spatial-temporal trends in ECSCs and characterizing access to emergency care using existing roadway networks to determine where gaps in care are occurring.

The results of this study demonstrate the presence of significant barriers to accessing emergency care in Brazil. Socioeconomic indicators in Brazil generally ascribe to a North-South macro-regional gradient with the worst indicators in the Northern parts of the country [29,30]. Healthcare access in the North region is recognized to be insufficient to meet the needs of the population and the region's challenges are well documented in the literature [29]. However, our findings demonstrated that the access issues are not only restricted to the North. Other portions of the country, especially peri-urban areas, are also facing challenges in the way of geographical barriers to accessing emergency care.

In the Northeast and Southeast, there were clusters of increasing trends in admissions that remained significant when overlapped with areas experiencing geographical barriers to care. There is also a small area in the state of Mato Grosso in the Midwest that displays a similar trend. These should be priority areas for policy makers. However, the underlying reasons for the patterns in the Northeast and the Southeast are likely different. The trend in the Northeast is mostly seen in the state of Bahia, one of the country's poorest states [31]. Although the state has seen recent economic growth, the growth is not distributed equally. Rather, municipalities in semiarid rural areas continue to trail in socioeconomic indicators [31]. The existence of a large number of small hospitals, with a low capacity to perform emergency care, in Bahia may be related to the increasing trend in ECSC admissions [7]. Similar trends are also seen in the Southeast region of Brazil. However, this region has some of the best indicators as it has the highest GDP per capita and its four states are the richest in the country [32]. Thus, the increasing trends flagged in the states of Minas Gerais, Rio de Janeiro, and Espírito Santo are likely due to the large population outpacing the capacity of the EM centers. Per the 2010 census, 42% of Brazil's inhabitants resided in the Southeast [33].

The South and some areas of the Midwest saw the opposite pattern in terms of ECSC admissions. In those areas, space-time analyses demonstrated predominately Cold Spots. Populations in these regions are smaller; only 7•4% of the country resides in the Midwest and 11•6% resides in the South [34]. Further, in the South, these trends are likely related to the favorable socioeconomic indicators as well as the region's strong healthcare network. The number of doctors by population is significantly higher in the South (2•06/1000) than in the North (0•9/1000) [35]. In the South, the Family Health Strategy, Brazil's community-based model of primary care delivery, covers 64•8% of households and the region shows the least between-quartile variation [36].

In contrast to the other four regions, the North did not show significant space-time clustering. However, gaps in access were relevant in other analyses. The North is covered by the Amazon Rainforest and over one fifth of the population lives in rural areas [29]. Many forested and rural areas have limited organized road systems [8]. Thus, difficulties with travel are common and many people rely on river transportation to reach city hospitals [37,38]. Our results demonstrated that, in this region, only 69.1% of the population had access to an EM hospital within 180 minutes. In contrast, in the United States, 98% of the population has access to an Emergency Department within 60 minutes [39]. This high degree of accessibility (97-99%) holds true even for states with a high proportion of inhabitants living in rural regions [39]. Previous work in Brazil showed a significant shortage of both small hospitals and high complexity centers in the North with accessibility indices close to zero [7].

Nearly 25% of the country's municipalities are presenting with an increasing trend in ECSC admissions and requiring long distances to reach emergency care. However, the challenges regarding access to emergency care within Brazil vary by region. In the North, the major challenge is the lack of a well-structured transportation network. Brazil has implemented several initiatives to address this problem such as primary care fluvial mobile units and multidisciplinary teams focusing on reducing transportation barriers [40,41]. Another approach to minimizing geographical barriers to reaching emergency facilities is to improve access to prehospital emergency care. Instituted in 2003 and continuing to expand, SAMU, Brazil's Mobile Emergency Medical Service, reached 53•9% of the population in 2009 and 70•9% of the population by 2012 [42,43]. In contrast to the transportation issues in the North, in the Northeast and Southeast regions, the major challenge is the distribution of health services. These findings and interpretations can help guide policy makers in making region-specific policy decisions to increase the emergency hospital coverage in the country. The regions highlighted in the states of Bahia, Minas Gerais, Espiríto Santo, Tocantins, and Amapá should be considered priority areas. The approach developed here can be used in other contexts or countries as the basis for evidence-based policy discussions.

Despite the use of innovative methods, the present work has some limitations. One limitation of this approach is that geolocation was not possible in all areas. In rural locations, the footpaths and unpaved roads are not always included in mapping projects and there can be problems with a lack of zip codes [40]. In urban regions, challenges arose when zip codes could not be precisely geolocated. Although geolocation was not possible in all areas, we still chose to analyze ECSC admissions in the whole country, in line with our objective to evaluate access to emergency care from a population- and health system-level perspective. We were successfully able to geolocate 94% of admissions (Supplementary Materials, Tables 1,2,&3).

Additionally, as we only used hospital-level and not EMS-level data or data from death certificates, we were unable to account for ECSCs that resulted in death prior to hospital presentation, resulting in selection bias. Further, in determining the catchment areas, our threshold of 180 minutes, although in accordance with the Lancet Commission recommendations, does not necessarily apply to all ECSCs [22]. Some conditions, like ST-elevation myocardial infarction or stroke, have individual well-established time-to-treatment guidelines. Lastly, as a metric for assessing time to care, our approach only assessed travel time and not time spent in a healthcare facility prior to being seen. We acknowledge that physically reaching a facility is different from receiving care and recognize that our approach does not account for all aspects of care delays and as result, is likely underestimating the problem.

To our knowledge, this is the first study evaluating the accessibility of emergency care in Brazil by characterizing space-time trends in ECSC admissions and distance to hospitals along established road networks. This analysis was made possible through the use of information contained within the Brazil Sistema Único de Saúde datasets, demonstrating the importance of national open access databases for research purposes. If other countries have the capacity to similarly offer publicly available health information, this approach could be used to characterize many aspects of access to care. We hope the results of our work will be used in discussions on future public health interventions surrounding hospital and prehospital infrastructure development.

## 5 Conclusions

The distribution of ECSCs in Brazil's five regions across time and space is highly heterogeneous. Significant geographical barriers to accessing emergency care exist in certain regions of Brazil. Challenges with access to healthcare in the North are well described in the literature but our results demonstrated that other regions of the country are also facing barriers. The findings from our innovative approach can be used by policymakers and health authorities to support discussions on addressing geographical barriers to emergency care in regions facing increasing trends in ECSC admissions. We hope our findings will inform debates on the location-allocation of health resources to better organize the health care network. Going forward, subsequent work should evaluate mortality data and target specific emergency conditions to gain further insight into how to improve the accessibility of emergency care in Brazil.

## Contributors

Julia Isaacson was responsible for conceptualization, visualization, writing – original draft, and writing – review & editing. Anjni Joiner was responsible for conceptualization, funding acquisition, supervision, visualization, and writing – review and editing. Arthi Kozhumam was responsible for conceptualization, writing – original draft, and writing – review & editing. Nayara Caruzzo was responsible for data curation, formal analysis, investigation, software, and writing – review & editing. Luciano de Andrade was responsible for data curation, formal analysis, investigation, software, and writing – review & editing. Pedro Iora was responsible for data curation, formal analysis, investigation, software, validation, and writing – review & editing. Dalton Costa was responsible for data curation, formal analysis, investigation, software, and writing – review & editing. Bianca Vissoci was responsible for data curation, project administration, and writing – review & editing. Marcos Sartori was responsible for data curation, formal analysis, investigation, software, and writing – review & editing. Thiago Rocha was responsible for conceptualization, formal analysis, methodology, project administration, software, validation, visualization, writing – original draft, and writing – review & editing. Joao Vissoci was responsible for conceptualization, formal analysis, funding acquisition, methodology, project administration, resources, supervision, visualization, validation, and writing – review & editing. Pedro Iora, Thiago Rocha, and Joao Vissoci, and were responsible for verification of the underlying data.

## Declaration of interests

This study was funded by the Duke Global Health Institute Artificial Intelligence Pilot Project.

## Data sharing statement

Data for this study was collected from open access sources as listed in Table 3. All data is publicly available. Table 4.

**Table 4:** Characteristics of municipality by Brazilian region, 2015-2019.

	**South**	**Southeast**	**Mid-West**	**Northeast**	**North**	**Brazil**
ECSCs Md (Q1;Q3)	138 (65;335•75)	132 (61;360•25)	89 (38;264)	108 (49;240)	100 (38;281)	118 (54;304)
# of EM hospitals median (min, max)	0 (0;36)	0 (0;158)	0 (0;50)	0 (0;52)	0 (0;34)	0 (0;162)
# of EM hospitals in non-zero municipalities median (min, max)	1 (1;36)	1 (1;158)	1 (1;50)	1 (1;52)	1 (1;34)	1 (1;162)
# of EM hospitals mean (+/- sd)	0•457 (1•742)	0•868 (5•35)	0•53 (3•48)	0•442 (2•510)	0•44 (2•28)	0•525 (3•50) ANOVA p = 0•026
# of EM hospitals in non-zero municipalities mean (+/- sd)	1•61 (3•02)	3•02 (10•8)	3•06 (7•91)	2•4 (5•46)	2•72 (2•78)	2•52 (7•62) ANOVA p = 0•15
# of inpatient beds/EM hospital median (min, max)	43 (27;988)	50 (30;1543)	26 (16;666)	28 (16;1075)	31 (20;561)	35 (16;1543)
Average distance to hospital in km (SD)	10•20 (12•68)	12•62 (18•28)	21•22 (27•87)	17•28 (22•99)	22•38 (33•17)	14•53 (21•00)
Percentage of population residing within 180 minutes of an EM hospital	99•4%	100%	93•3%	93•2%	69•1%	95•3%

## Editor note

*The Lancet Group takes a neutral position with respect to territorial claims in published maps and institutional affiliations.*

## Appendix Supplementary materials

Image, application suppl1

Image, application supp2
