# Supplementary material for: Emergency Care Sensitive Conditions in Brazil: A Geographic Information System Approach to Timely Hospital Access
Source: Lancet Reg Health Am. 2021 Sep 10;4:100063. doi: 10.1016/j.lana.2021.100063 (PMC9903578; doi:10.1016/j.lana.2021.100063)
Supplement: Supplementary file 1 [file mmc1.docx]

Supplementary Materials

**Table 1. Quality of Geocoding (Urban – Rural)**

| **Characteristic** | **Geocoded** | **Not geocoded** | **Total** |
| --- | --- | --- | --- |
| Type of municipality (rural - urban) |  |  |  |
| Intermediary close to urban area | 819,130 (99%) | 7,706 (0.9%) | 826,836 (100%) |
| Intermediary remote | 55,916 (98%) | 1,242 (2.2%) | 57,158 (100%) |
| Rural close to urban area | 1,721,063 (98%) | 27,627 (1.6%) | 1,748,690 (100%) |
| Rural Remote | 154,820 (98%) | 2,491 (1.6%) | 157,311 (100%) |
| Only the state identified | 57,714 (99%) | 349 (0.6%) | 58,063 (100%) |
| Urban | 6,827,515 (92%) | 603,868 (8.1%) | 7,431,383 (100%) |
| **Total** | **9,636,158 (94%)** | **643,283 (6.3%)** | **10,279,441 (100%)** |

Overall, we were able to geocode a high number (94%) of ECSC admissions from 2015-2019. The quality of geocoding was the poorest in urban areas, where 8.1% of admissions were not geolocated. In all other categories of municipality, we were able to geolocate at least 98% of admissions.

**Table 2. Distribution of ECSC Admissions by Type of Municipality (Urban – Rural)**

| **Characteristic** | **Overall, N = 10,279,441^1^** | **Intermediary close to urban areas, N = 826,836^1^** | **Intermediary Remote, N = 57,158^1^** | **Rural close to urban area, N = 1,748,690^1^** | **Rural Remote, N = 157,311^1^** | **Only state reported, N = 58,063^1^** | **Urban, N = 7,431,383^1^** |
| --- | --- | --- | --- | --- | --- | --- | --- |
| **States** |  |  |  |  |  |  |  |
| Acre | 27,472 (0.3%) | 2,860 (0.3%) | 4,364 (7.6%) | 3,542 (0.2%) | 1,317 (0.8%) | 0 (0%) | 15,389 (0.2%) |
| Alagoas | 121,550 (1.2%) | 6,118 (0.7%) | 0 (0%) | 27,229 (1.6%) | 0 (0%) | 0 (0%) | 88,203 (1.2%) |
| Amapá | 25,697 (0.2%) | 460 (<0.1%) | 926 (1.6%) | 597 (<0.1%) | 1,209 (0.8%) | 0 (0%) | 22,505 (0.3%) |
| Amazonas | 131,876 (1.3%) | 2,282 (0.3%) | 8,098 (14%) | 5,143 (0.3%) | 11,571 (7.4%) | 0 (0%) | 104,782 (1.4%) |
| Bahia | 624,775 (6.1%) | 82,742 (10%) | 1,104 (1.9%) | 201,279 (12%) | 18,278 (12%) | 0 (0%) | 321,372 (4.3%) |
| Ceará | 416,548 (4.1%) | 37,703 (4.6%) | 0 (0%) | 122,291 (7.0%) | 0 (0%) | 0 (0%) | 256,554 (3.5%) |
| Distrito Federal | 111,583 (1.1%) | 0 (0%) | 0 (0%) | 0 (0%) | 0 (0%) | 55,821 (96%) | 55,762 (0.8%) |
| Espirito Santo | 192,689 (1.9%) | 37,279 (4.5%) | 0 (0%) | 43,254 (2.5%) | 0 (0%) | 0 (0%) | 112,156 (1.5%) |
| Goiás | 329,507 (3.2%) | 27,319 (3.3%) | 2,265 (4.0%) | 43,289 (2.5%) | 0 (0%) | 0 (0%) | 256,634 (3.5%) |
| Maranhão | 303,780 (3.0%) | 41,106 (5.0%) | 2,014 (3.5%) | 95,691 (5.5%) | 21,375 (14%) | 0 (0%) | 143,594 (1.9%) |
| Mato Grosso | 176,402 (1.7%) | 15,101 (1.8%) | 13,470 (24%) | 13,897 (0.8%) | 23,292 (15%) | 0 (0%) | 110,642 (1.5%) |
| Mato Grosso do Sul | 153,619 (1.5%) | 12,330 (1.5%) | 3,075 (5.4%) | 15,166 (0.9%) | 8,331 (5.3%) | 43 (<0.1%) | 114,674 (1.5%) |
| Minas Gerais | 1,201,345 (12%) | 120,267 (15%) | 6,108 (11%) | 270,555 (15%) | 11,939 (7.6%) | 0 (0%) | 792,476 (11%) |
| Pará | 381,096 (3.7%) | 21,463 (2.6%) | 10,314 (18%) | 61,087 (3.5%) | 25,918 (16%) | 208 (0.4%) | 262,106 (3.5%) |
| Paraíba | 168,710 (1.6%) | 17,390 (2.1%) | 0 (0%) | 40,197 (2.3%) | 0 (0%) | 0 (0%) | 111,123 (1.5%) |
| Paraná | 896,673 (8.7%) | 98,579 (12%) | 0 (0%) | 200,049 (11%) | 0 (0%) | 0 (0%) | 598,045 (8.0%) |
| Pernambuco | 450,307 (4.4%) | 49,770 (6.0%) | 155 (0.3%) | 58,473 (3.3%) | 108 (<0.1%) | 0 (0%) | 341,801 (4.6%) |
| Piaui | 191,423 (1.9%) | 31,300 (3.8%) | 2,469 (4.3%) | 69,313 (4.0%) | 13,992 (8.9%) | 0 (0%) | 74,349 (1.0%) |
| Rio de Janeiro | 542,207 (5.3%) | 18,605 (2.3%) | 0 (0%) | 16,850 (1.0%) | 0 (0%) | 0 (0%) | 506,752 (6.8%) |
| Rio Grande do Norte | 120,484 (1.2%) | 17,260 (2.1%) | 0 (0%) | 31,528 (1.8%) | 0 (0%) | 0 (0%) | 71,696 (1.0%) |
| Rio Grande do Sul | 835,569 (8.1%) | 46,039 (5.6%) | 123 (0.2%) | 172,357 (9.9%) | 503 (0.3%) | 66 (0.1%) | 616,481 (8.3%) |
| Rondônia | 92,848 (0.9%) | 8,069 (1.0%) | 1,338 (2.3%) | 10,275 (0.6%) | 7,242 (4.6%) | 0 (0%) | 65,924 (0.9%) |
| Roraima | 27,906 (0.3%) | 0 (0%) | 0 (0%) | 5,222 (0.3%) | 3,436 (2.2%) | 0 (0%) | 19,248 (0.3%) |
| Santa Catarina | 463,478 (4.5%) | 24,696 (3.0%) | 0 (0%) | 135,520 (7.7%) | 0 (0%) | 1,925 (3.3%) | 301,337 (4.1%) |
| São Paulo | 2,165,624 (21%) | 90,600 (11%) | 0 (0%) | 80,760 (4.6%) | 0 (0%) | 0 (0%) | 1,994,264 (27%) |
| Sergipe | 61,950 (0.6%) | 9,177 (1.1%) | 0 (0%) | 12,813 (0.7%) | 0 (0%) | 0 (0%) | 39,960 (0.5%) |
| Tocantins | 64,323 (0.6%) | 8,321 (1.0%) | 1,335 (2.3%) | 12,313 (0.7%) | 8,800 (5.6%) | 0 (0%) | 33,554 (0.5%) |
| **Admissions geocoded** | | | | | | | |
| Geocoded | 9,636,158 (94%) | 819,130 (99%) | 55,916 (98%) | 1,721,063 (98%) | 154,820 (98%) | 57,714 (99%) | 6,827,515 (92%) |
| Not geocoded | 643,283 (6.3%) | 7,706 (0.9%) | 1,242 (2.2%) | 27,627 (1.6%) | 2,491 (1.6%) | 349 (0.6%) | 603,868 (8.1%) |
| ***^1^ Statistics presented: n (%)*** | | | | | | | |

This is an extension of table 1, demonstrating that the distribution of ECSC admissions by type of municipality varies by state.

**Table 3. Quality of Geocoding by Brazilian Region**

| **Regions** | **Geocoded** | **Not geocoded** | **Total** |
| --- | --- | --- | --- |
| Midwest | 752,012 (98%) | 19,099 (2.5%) | 771,111 (100%) |
| Northeast | 2,413,773 (98%) | 45,754 (1.9%) | 2,459,527 (100%) |
| North | 735,794 (98%) | 15,424 (2.1%) | 751,218 (100%) |
| Southeast | 3,655,772 (89%) | 446,093 (11%) | 4,101,865 (100%) |
| South | 2,078,807 (95%) | 116,913 (5.3%) | 2,195,720 (100%) |
| Total | 9,636,158 (94%) | 643,283 (6.3%) | 10,279,441 (100%) |

The majority (94%) of ECSC admissions from 2015-2019 were successfully geolocated. The quality of geolocation was poorest in the more urban regions of the country, the Southeast and South, where 11% and 5.3% of admissions, respectively, could not be geolocated. In the North, Midwest, and Northeast, we successfully geolocated 98% of admissions over a five-year period.
