# Supplementary material for: Emergency Care Sensitive Conditions in Brazil: A Geographic Information System Approach to Timely Hospital Access
Source: Lancet Reg Health Am. 2021 Sep 10;4:100063. doi: 10.1016/j.lana.2021.100063 (PMC9903578; doi:10.1016/j.lana.2021.100063)
Supplement: Supplementary file 2 [file mmc2.docx]

Editor Note:

“*This translation in Portuguese was submitted by the authors and we reproduce it as supplied. It has not been peer reviewed. Our editorial processes have only been applied to the original abstract in English, which should serve as reference for this manuscript.*”

Resumo

Antecedentes

Os benefícios do tratamento para diveras condições de saúde são dependentes de tempo. O ônus dessas condições sensíveis aos cuidados de emergência (ECSCs – sigla em inglês) é especialmente alto em países de baixa e média renda. Nosso objetivo foi analisar as tendências geoespaciais dos ECSCs e caracterizar as disparidades regionais no acesso aos cuidados de emergência no Brasil.

Métodos

A partir de conjuntos de dados disponíveis publicamente, extraímos dados de pacientes com códigos de CID-10 associados a ECSCs, bem como sobre as instalações de emergência do país a partir de 2015-2019. Usando ArcGIS, OpenStreetMap e WorldPop, criamos polígonos correspondentes a 180 minutos de distância de carro de cada hospital. Usamos então o ArcGIS para caracterizar as tendências espaço-temporais nas admissões ECSCs e para completar uma matriz de Origem-Destino para determinar a rota mais curta entre a residência do paciente até o hospital mais próximo.

Conclusões

Havia 1362 municípios assinalados como "pontos quentes", áreas com um alto volume de ECSCs. Desses, 69,7% estavam a mais de 180 minutos (171 km) das instalações de emergência mais próximas. Estes municípios estavam localizados principalmente nos estados de Minas Gerais, Bahia, Espiríto Santo, Tocantins e Amapá. Na região Norte, apenas 69,1% da população residia dentro de 180 minutos de um hospital de emergência.

Interpretações

Existem barreiras geográficas significativas ao acesso aos cuidados de emergência em certas áreas do Brasil, especialmente em áreas periurbanas e na região Norte. Uma limitação desta abordagem é que a geolocalização não foi possível em algumas áreas e, portanto, provavelmente estamos subestimando o ônus de um acesso inadequado. Os trabalhos subseqüentes devem avaliar os dados de mortalidade da CSCE.

Financiamento

Este estudo foi financiado pelo Projeto Piloto de Inteligência Artificial do Duke Global Health Institute.
